# Repairing Behaviors of Cracked Steel Plates Based on Bolted Fiber-Reinforced Polymer Plates

**DOI:** 10.3390/ma16206773

**Published:** 2023-10-19

**Authors:** Jie Liu, Haobo Wang, Yang Wei, Daguang Han, Yunfei Xiang

**Affiliations:** 1 College of Civil Engineering, Nanjing Forestry University, Nanjing 210037, China; jieliu@outlook.com (J.L.); whb20230426@163.com (H.W.); yfxiang@njfu.edu.cn (Y.X.); 2 School of Civil Engineering, Southeast University, Nanjing 210096, China; daguanghan@seu.edu.cn

**Keywords:** cracked steel plate, fiber-reinforced polymer, repair, stress, pre-tightening force

## Abstract

The use of FRP materials to repair cracked/damaged steel structures has gradually been adopted by researchers. This paper investigates the repairing effect of bolted FRP plates for cracked steel plates based on experimental and numerical simulation methods. In the experimental investigation, the tensile strengths of six specimens, including three repaired specimens and three pure cracked steel specimens, were evaluated. The test outcomes indicated that the bolt repairing method significantly enhanced the tensile strengths of the cracked steel plates. As an example, the failure of a pure steel plate with a 1 mm width crack occurred at 813 N, whereas after being repaired, a tensile strength of 1298 N was observed. Based on finite element (FE) analysis, the influence of bolt preloads and interfacial friction coefficients were verified. The stress-relative ratio for specimens was contingent on the bolt preload magnitude and gradually decreased as the preload was augmented. By exploring the repairing effect for varied friction coefficients, it was concluded that using a higher bolt preload can aid in eliminating the performance discrepancy of the overall component caused by interface treatment errors.

## 1. Introduction

Steel elements are extensively employed in diverse fields, such as aerospace engineering, civil engineering, marine engineering, etc. During practical application, owing to external loads and working environment conditions, steel members are inevitably prone to damage, cracks, and even failures [1,2,3]. In practical applications, preventing cracks from spreading and evolving into through-thickness cracks is of the utmost importance, particularly in the fields of bridge engineering, aerospace engineering, transportation pipelines, and pressure vessels [4,5,6]. Recently, fiber-reinforced polymer (FRP) has been increasingly used in the aviation industry, civil engineering, and energy industry sectors because of its light weight, high strength, super fatigue resistance, and corrosion resistance [7,8,9,10,11]. The technology of repairing/reinforcing structures with FRP materials has attracted more and more scholars’ attention [12,13].

Typically, there are two techniques for repairing steel structures with FRP, namely external bonding and bolting methods. For the bonding method, high-performance bonding materials are employed to affix the FRP material onto the surface of the base material, allowing both to distribute the force. On the other hand, the bolt connection method utilizes bolts to establish a connection between the FRP and the base structure, resulting in the FRP material being secured together with the structure as a whole [14,15,16,17,18]. For actual reinforcement, considering the low durability and high-temperature sensitivity of the bonding material and the fragility of the interface, the bolted connection method can be regarded as a significant practical and competitive reinforcement method.

The study of bolted FRP-reinforced/repaired structures has been an area of research interest. Hai and Mutsuyoshi [19] researched the structural behavior of double-lap joints composed of steel splice plates bolted and bonded to pultruded hybrid CFRP/GFRP laminates, revealing that the bolted joint’s failure strength and failure mode were predominantly influenced by the bolt-end distance. Feroldi and Russo [20] conducted experimental research on the structural performance of FRP beam–column–plate bolted connections by determining the initial stiffness and ultimate bending capacity for each connection and the rotation in relation to ultimate strength. These results were then compared to numerical predictions obtained through commercial code utilizing finite element analysis. Sweedan et al. [21] investigated the interfacial behavior of mechanically anchored FRP laminates for strengthening steel beams. The failure of test components was primarily controlled by using a combination of various mechanisms, including bearing in FRP laminates, bending in connecting bolts, folding in washers, and tearing of FRP laminates. Later, Satasivam et al. [22] conducted an experimental study on the composite actions present in steel-FRP composite beam systems utilizing novel blind bolt shear connections. They quantified the shear stiffness, also known as the slip modulus, of the shear connection by subjecting steel-FRP joints to tension loads. Abdelkerim et al. [23] investigated the static and fatigue performance of FRP bolted joints and proposed a new hybrid steel-FRP bolt to prolong the fatigue life of the composite joints. Recently, Olivier et al. [24] investigated the feasibility of bolted connectors in hybrid FRP-steel structures via experimental analysis. The authors confirmed the great application prospect of the bolted FRP-steel composite structure for engineering structures and proposed novel preloaded connectors injected with steel-reinforced resin for the bolted connections. The above-mentioned researchers undertook extensive studies on the performance of FRP-steel bolted connections. These studies demonstrate the enormous potential of FRP bolted repair/reinforcement of steel components.

However, the existing research mainly focuses on the conventional reinforcement connection performance of typical components, and there is little research on FRP bolted repair of cracked steel specimens in engineering. The research results introduced previously have proven that FRP bolted technology has great potential for repairing/strengthening steel components. The specific efficiency and influencing factors of this repair technology need further research. Therefore, this study investigated the repair of cracked steel plate specimens using the bolt connection method through experimental and simulating analysis. It comprehensively analyzed the effects of crack size and bolt preload on the repairing performance, providing a solid scientific basis for the application of FRP bolted steel specimens.

## 2. Repair Experiment

### 2.1. Materials

In this study, 3 mm thick Q355b steel, with a yield strength and Young’s modulus measuring 364 MPa and 203 GPa, respectively, according to the manufacturer’s specifications, was the primary material used for constructing the steel plates. The outer contour size of the slotted steel plate was 150 mm × 100 mm. Prefabricated at specified positions, two bolt holes featuring a 7 mm diameter were included on each steel plate. For this research study, 6 mm diameter stainless-steel screw arbors were utilized. The Young’s modulus and shear strength of the stainless-steel bolts were provided by the manufacturer, measuring 202 GPa and 645 MPa, respectively. Tabulated in Table 1 are the material properties adopted throughout the research, including those of the pultruded GFRP plates, reinforced adhesive, and steel. These GFRP plates, possessing a thickness of 3 mm, were constructed from E-glass fibers and unsaturated polyester resin.

### 2.2. Manufacturing and Repairing Mechanism

**Figure 1:**
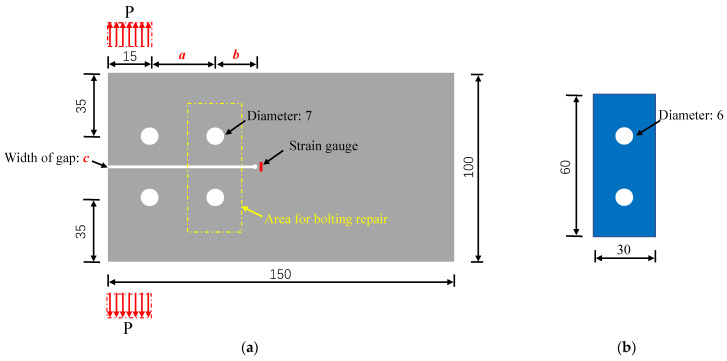
Geometry and load graph of the FRP steel bolted repaired specimen (unit: mm). (**a**) Cracked steel plate. (**b**) FRP plate.

Table 2 presents information regarding the tested specimens—consisting of three repaired specimens (type I, II, and III)—and three reference specimens (reference I, II, and III), which were pure cracked steel plates. To construct the repaired specimens, a cracked steel plate, two FRP plates, and two steel bolts were integrated into their structural design, as illustrated by the geometric features shown in Figure 1 and Table 2. It is worth noting that no initial preload (i.e., pre-tightening force) was imposed on the bolts of the three bolted repaired specimens analyzed in this study. To analyze the strain development throughout the quasi-static tensile test, strain sensors were placed on the steel plates’ surface adjacent to the crack tip. The test was carried out using displacement control mode at a constant rate of 1 mm/min. Additionally, to monitor the stress changes in the region around the crack tip during the stretching process, a strain gauge was positioned 3 mm in front of the tip, as depicted in Figure 1. Furthermore, the tensile strength and failure characteristics of the six specimens were obtained based on the quasi-static experiments, as illustrated in Table 2. 

**Figure 2:**
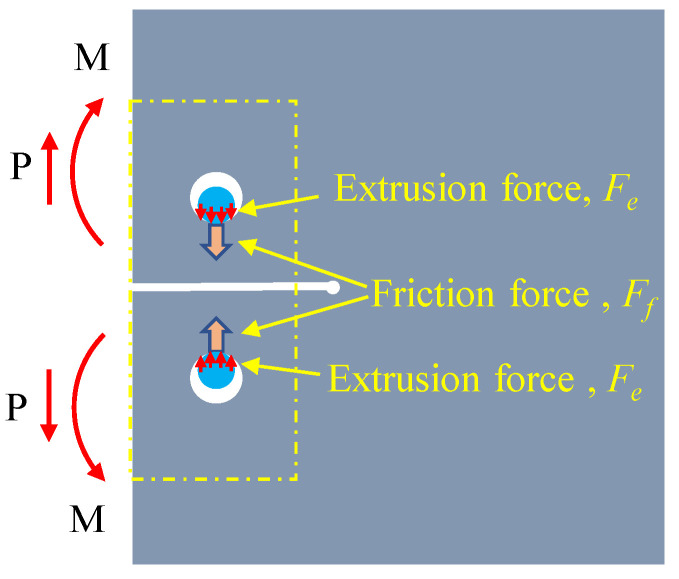
Load transfer diagram of the repaired specimen.

**Figure 3:**
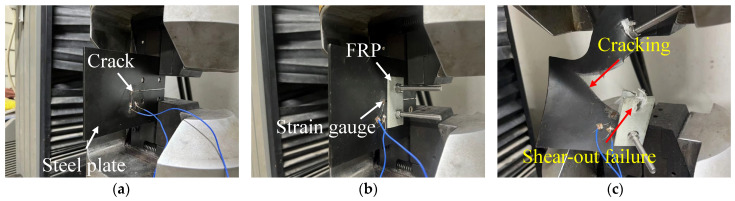
Test setup and failure modes. (**a**) For reference specimen. (**b**) For repaired specimen. (**c**) Composite failure modes.

Under tensile loading *P*, the repaired specimen experienced shear force (*P*) and a bending moment (*M*) in the left section proximate to the reinforcement area (comprising the upper and lower sections). Meanwhile, the local bolted area was subjected to surface friction (*F_f_*) between the FRP and steel plate as well as extrusion force (*F_e_*) between the screw and steel plate screw hole wall (which may occur only after slippage of the FRP and steel plate), as shown in Figure 2. Based on the force transmission mechanism in Figure 3, it is easy to conclude that the repaired specimen utilized the new force transmission path (offered by the bolted combination area) to transfer a portion of the internal load. By using this technique, the stress on the cracked area can be reduced, ultimately leading to the safeguarding and reinforcement of the cracking region. The greater the internal loads transferred, the more effective the repair/reinforcement will prove to be.

## 3. Repair Performance Analysis

### 3.1. Testing Results

**Figure 4:**
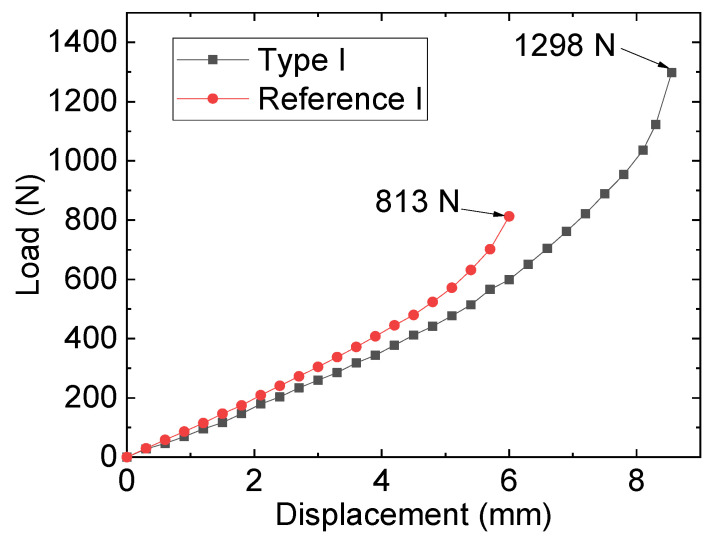
Load–displacement curves of specimens type I and reference I.

Figure 3 plots the tests and failure modes of the specimens, and the results of the specimens labeled as reference I, II, and III in Table 2 reveal that the width of the steel plate crack affected the specimen’s failure strength, with the narrower crack width resulting in lower failure strength of the specimen. For instance, reference I had a tensile strength of 813 N, whereas 1204 N was adopted by reference III. A similar trend was observed in types I, II, and III. This demonstrated that the crack width exerted a vital influence on the specimen’s cracking strength. By comparing the reference specimen and its corresponding repaired specimen, it was found that the adopted repairing method could significantly improve the tensile specimen of the cracked steel plates. For example, the failure of the specimen for reference I occurred at 813 N, whereas 1298 N of the tensile specimen was adopted by the specimen of repairing type I. For the reference specimens, during the loading process, because of the stress concentration effect in the crack area, the reference specimen cracked rapidly, and as the load continued to be applied, the crack gradually expanded until the overall failure of the plate. However, the damage of the repaired specimens exhibited distinct characteristics: during the initial loading stage, due to the bolting and anchoring effect of the FRP plate, the load-bearing capacity of the repaired specimen was mainly supported by the FRP plate. As the external load gradually increased to a certain value, shear-out failure of the FRP plate occurred and eventually caused the failure of the steel plate due to cracking. Figure 4 plots the load–displacement curves of specimens type I and reference I.

### 3.2. Finite Element Models

**Figure 5:**
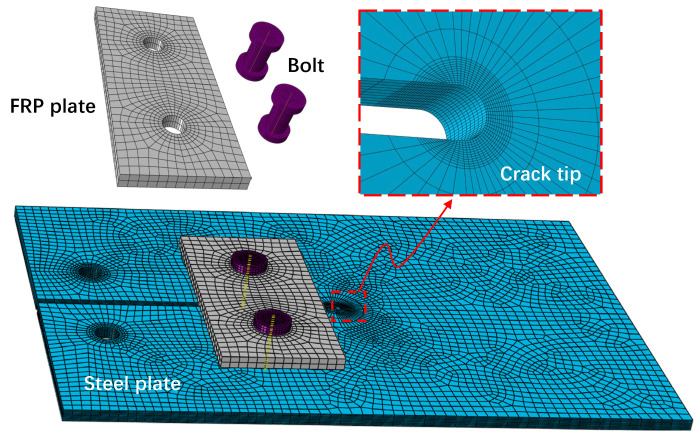
Repaired FE model.

Three-dimensional (3-D) finite element (FE) analysis using ABAQUS was employed to investigate the repair efficiency of the bolted FRP method for cracked steel plates. The bolted repaired model comprised one cracked steel plate, two FRP plates, and two bolts, while the steel plate and steel bolt were assumed to be homogeneous and linearly elastic, based on Table 1 values. Concurrently, the orthotropic linear elastic material model for the FRP laminate plate referenced the longitudinal, transverse, and thickness modulus values in Table 1. The sizes of the steel, FRP, and bolt models were established based on the actual sizes of the bolted repaired specimens. The steel plate’s screw hole diameter was 7 mm, and the FRP screw hole diameter was 6 mm, with a 6 mm diameter screw arbor and 9 mm outer diameter nut. The steel plate, FRP plate, and bolt component (i.e., nuts and screw arbors) were simulated using eight-node linear brick elements (C3D8R) suitable for complex nonlinear analyses of bolted specimens, as evidenced in Mandal and Anupam [25] and Yang et al. [26]. Moreover, wedge elements were implemented to mesh the prefabricated crack’s tip region and the center core portion of the bolting zones of the steel plates. Finer wedge elements were applied near the bolt holes of the FRP plates due to the high stress concentration. Each screw hole was divided into 32 units along the perimeter. The mesh size of the overall model was approximately 2 mm, while hexahedral elements represented the crack area of the steel plate with a mesh size of 0.1 mm to capture the stress distribution of the crack tip accurately. Figure 5 illustrates one of the repaired FE models’ layouts, and the model’s constraints were established according to actual stress and deformation conditions.

An in-built master–slave algorithm was used to define the contact relationships among various components of the repaired model. This study encompassed different contact behaviors, such as the contact between the bolt and FRP, the contact between the bolt and the steel plate, and the contact between the steel and FRP. These behaviors were primarily characterized by their tangential and normal properties. The contact behaviors in the normal direction were referred to as “hard contact” in this study, while the contact behaviors in the tangential direction were expressed through the application of “Coulomb friction force”. The finite sliding surface-to-surface discretization method was utilized to simulate the relative sliding of different components and to prevent stress concentration. McCarthy et al.’s [27] coefficients of friction of 0.1 and 0.3 for the bolt-to-FRP and steel-to-FRP interfaces, respectively, were adopted. 

**Figure 6:**
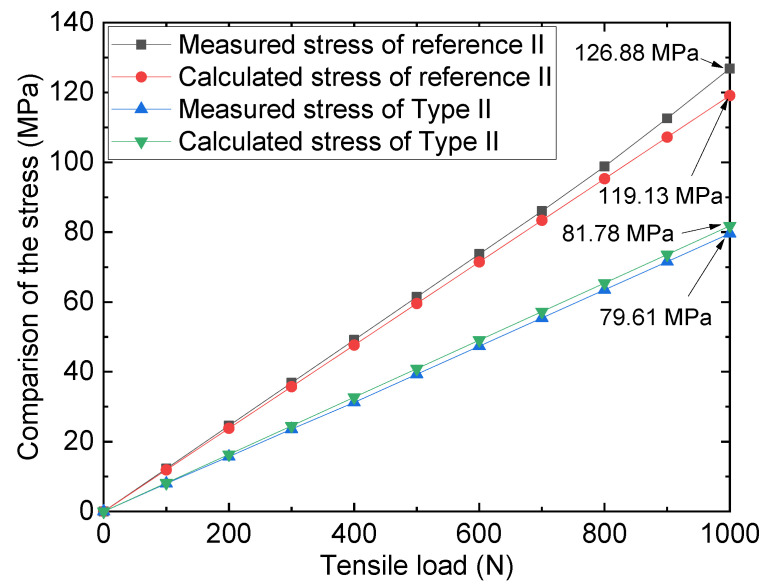
Comparison of the stresses of tests and FE models.

To validate the accuracy of the FE model method, the results from the 3-D FE model of the repaired specimen for type II (repaired by FRP plates without preload) and reference II (pure steel plate with 2 mm crack) were compared with the results from experiments. Figure 6 shows the comparison of the stresses of the measuring point of the crack tip (the measuring location of the strain gauges is shown in Figure 1) under different tensile loads. Upon comparison of the 3-D FE simulation analysis and experimental test stress values, it can be observed that the results are substantially consistent. For instance, the monitored stress level for the reference specimen under a 1000 N load was 126.88 MPa, whereas the model stress for the same load was 119.13 MPa, with a relative error of 5.5%. Furthermore, for the repaired specimen, the experimental stress under a 1000 N load was 79.61 MPa, whereas the corresponding model analysis stress level was 81.78 MPa, with the relative error amounting to roughly 2.7%. The comparison outcomes demonstrate that the 3-D FE analysis approach employed can effectively and precisely capture the loading information of the actual specimen. 

### 3.3. Stress Distribution of Reference II

**Figure 7:**
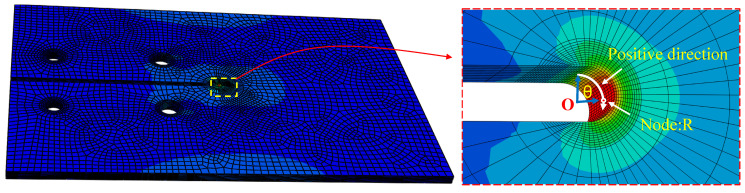
Stress distribution and location of calculated nodes.

Figure 7 exemplifies the stress distribution of reference II, which was subjected to a tensile force of 1000 N. The stress cloud diagram reflects significant stress concentration in the cracking tip, thereby suggesting that the specimen’s failure under tensile load was initiated in the crack tip area. To facilitate a thorough analysis of the stress distribution within the fracture tip region, the local polar coordinate system of the tip area was established, and the stress values of the edge nodes in this region were extracted. Specifically, the coordinate origin was defined as the center of the gap area (point O in Figure 7), while the middle plane of the crack gap served as the coordinate plane.

**Figure 8:**
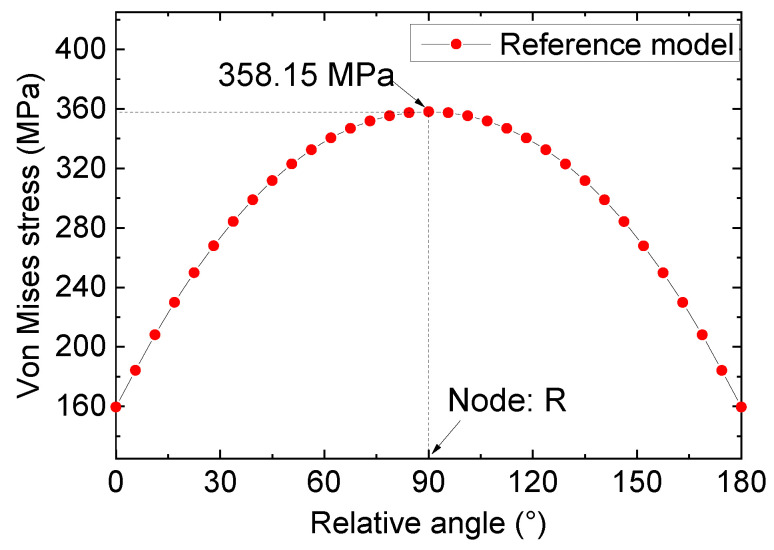
Stress distribution of the crack tip nodes for the reference model.

Drawing on the calculated path’s definition in Figure 7, the stress of crack tip nodes could be ascertained and are depicted in Figure 8. A detailed analysis of Figure 8 reveals substantial variation in the stress value at the crack tip with the azimuth angle’s augmentation. Specifically, for 0° < θ < 90°, the stress steadily increased with the increase in angle, and an incremental increase in the stress value was observed with the augmentation in the azimuth angle’s magnitude. For 90° < θ < 180°, the stress value displayed an opposite trend, with a gradual decrease in the stress values observed alongside an increase in azimuth angle. Notably, for the node (referred to as node “R”) with an azimuth angle of 90°, the stress value reached its maximum of 358.15 MPa. According to the stress distribution law of the crack notch, node R was used as the stress control point, and the subsequent analysis was based on the stress information of this node.

**Figure 9:**
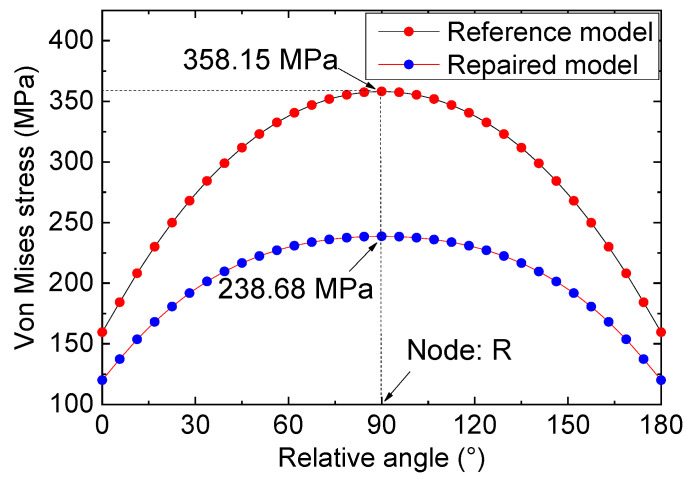
Comparison of the stress distribution of the repaired and reference model.

Figure 9 illustrates a stress comparison between the type II repaired specimen and the reference I specimen under a tension of 1000 N. Upon examining the data from Figure 9, it becomes apparent that the variation in the stress at the crack tip following repairing/strengthening with double-sided FRP plates mirrors that of the reference specimen. Nevertheless, upon scrutinizing the joint stresses of the two models at identical positions (for instance, the node, R), it becomes evident that the bolt repair of the FRP plates effectively mitigates the stress concentration at the crack tip in the specimen. As a consequence, the extreme stress level occurring near the tip following strengthening measures amounts to 238.68 MPa, which is significantly lower than the value of the reference specimen model (358.15 MPa), and the reduction range of 119.47 MPa was calculated accordingly.

### 3.4. Effect of Preload of the Bolts

**Figure 10:**
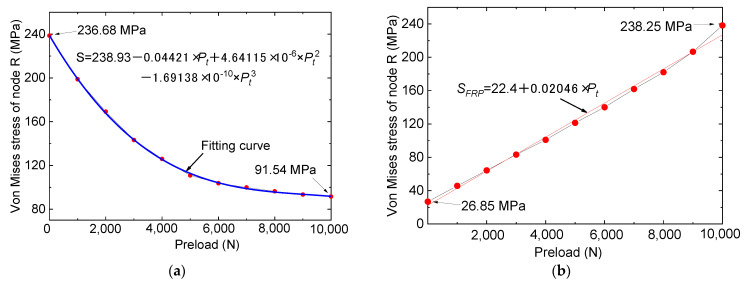
Comparison of the stresses for different preload conditions. (**a**) Stresses of node R. (**b**) Stresses of FRP plates.

It is worth noting that the tested specimens were manufactured without preload of the bolts; therefore, to investigate the pre-tightening effect of the bolts on the repaired specimens, FE analysis was carried out. To effectively simulate the pre-tightening effects of bolts on repairing efficiency, researchers adopted the in-built Bolt Load option in ABAQUS. Figure 10a offers an insightful comparison of the stresses of node R in the repaired specimen with various preload conditions under a 1000 N tensile load. It is essential to mention that the reinforced specimens shared the same type II steel plate. The valuable insights in Figure 8 demonstrate that the pre-tightening force of bolts played a crucial role in influencing the stress magnitude within a specimen’s crack area. As the pre-tightening force increases, the stress levels within the crack area gradually trend upwards. For instance, for specimens possessing 5000 N preloads, the maximum stress values at point R approximate 110.84 MPa, yet when the preload is augmented to 10,000 N, the maximum stress reduces to 91.54 MPa. The change in the von Mises stress for node R can be modeled kinetically with a cubic function curve, as Figure 10a shows, where *S* is the von Mises stress of node R and *P_t_* is the pre-tightening force of the bolts.

Figure 10b plots the comparison of the stresses of FRP plates for different preload conditions. From Figure 10b, it is discovered that, despite the effectiveness of bolt pre-tensioning in reducing stress concentration in the steel plate crack area, it inadvertently leads to stress concentration within the FRP plate itself, notably in the bolt hole region. Stress concentration in the FRP plate stems from two factors: firstly, the robust pre-tensioning action of the bolts, whereby the FRP plate bears more tensile load, and secondly, the initial pre-tensioning of the bolts increases the stress in the hole of the bolt. However, despite the stress concentration of the FRP plates, the magnitude of the forces involved does not surpass the failure strength of the FRP material itself. For instance, the stress threshold for the FRP plate in the 10,000 N preloaded specimen was about 238.25 MPa, which is considerably smaller than the failure strength of the FRP plate. Therefore, it may be deduced that within the predefined preloading limit (preloading force should not exceed 10,000 N), a higher preloading force will help repair and reinforce cracks in steel plates.

### 3.5. Effect of Crack Size

**Figure 11:**
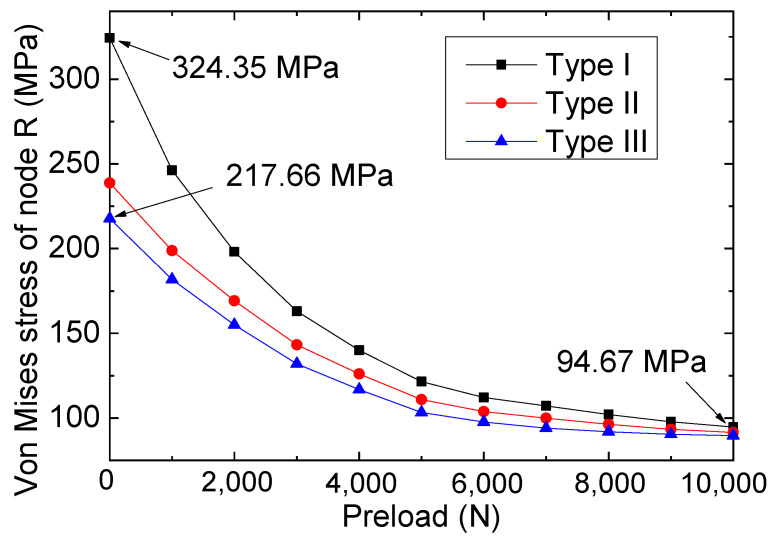
Stresses for node R for the three repaired models.

Previous research [28,29] has established the significance of crack size (mainly referring to the crack width) in defining the fracture performance of steel components. As a result, we conducted an analysis of repair/reinforcement based on FRP materials for steel plates with varying crack widths while simultaneously scrutinizing the impact of bolted connection repair under different pre-tightening forces on the failure performance of the specimens. Based on the test results for the repaired specimens in Table 2, the tensile strengths of the joints were significantly influenced by the crack widths, with a narrower crack width implying larger tip stress intensity. Figure 11 plots the stress of node R for the repaired model with different crack widths under a 1000 N tensile load. Based on the results in Figure 11, the stress of the crack tips was found to be significantly affected by the crack widths. For instance, for the type I specimen (1 mm width crack prefabricated in the steel plate) with 0 N pre-tightening force, the stress of node R was calculated as 324.35 MPa, whereas a stress of 217.66 MPa was found for type III. In addition, when the larger pre-tightening force (for example, 10,000 N) was applied through the bolts, the reinforcement effect of FRP on the steel plate was quite prominent, so the stresses at the crack tips of steel plates with different crack widths were close to each other; for example, the stresses of the three types of specimens were 94.67 (type I), 91.54 (type II), and 89.67 MPa (type III).

### 3.6. Effect of Interfacial Friction Coefficient

**Figure 12:**
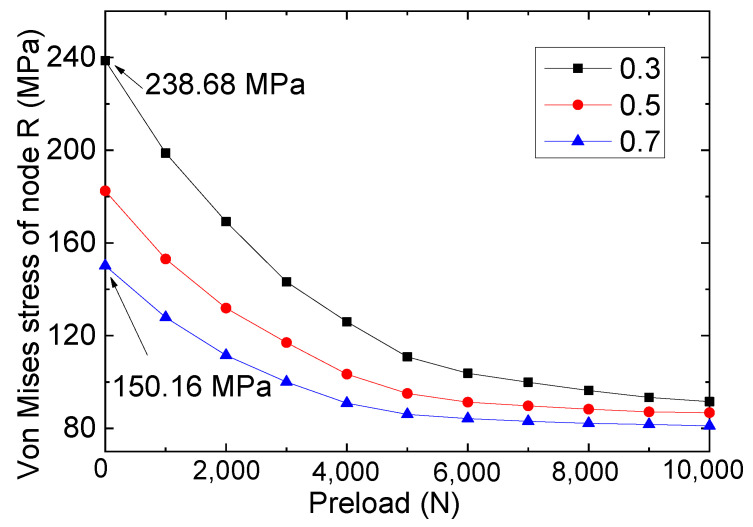
Stresses of node R for different friction coefficients.

Taking into account that the bolted repaired specimens studied previously were based on a coefficient of friction of 0.3 for the steel-to-FRP interfaces, this coefficient was confirmed by a study on bolted structures on conventional surfaces [27]. However, considering that surface treatment can be used to change the friction properties of a steel structure during the repair/strengthening process, it is necessary to investigate the effect of the friction coefficient of the interface on the mechanical performance of bolted repair components. Therefore, to explore the impact of the steel-FRP interfacial friction characteristics of type II repaired specimens on its tensile performance, a study on the mechanical properties of the specimens under varying friction coefficients was conducted. Three friction coefficients of 0.3, 0.5, and 0.7 were adopted, and the corresponding stresses of node R for each repaired specimen under a 1000 N tensile load are plotted in Figure 12. 

Based on Figure 12, the mechanical performance of the specimen will be affected by the interface friction coefficient. For example, for the repaired specimen under the same preload (for example, a preload of 5000 N), as the interface friction coefficient increased (from 0.3 to 0.7), the stress of node R gradually decreased from 110.84 to 86.03 MPa accordingly, indicating that a larger interface friction coefficient is more conducive to the unloading of the steel plate, which means that a larger interface friction coefficient of FRP-steel plates is more conducive to repair the cracked steel plates. Therefore, for the application of bolted FRP plates to repair cracked steel plates, it is advisable to increase the friction coefficient of the interface as much as possible through surface treatment.

In addition, by examining the data presented in Figure 12, it is evident that the performance of the repaired specimens was significantly influenced by changes in the friction coefficient at lower preloads (for preloads below 4000 N). For instance, at a preload of 1000 N, the analysis results for the three friction coefficients were 198.78 (0.3), 153.06 (0.5), and 125.85 MPa (0.7), respectively. Yet, for the working conditions featuring higher preloads (such as when the preloads were 10,000 N), closed values of 91.54 (0.3), 86.75 (0.5), and 81.06 MPa (0.7) were calculated. This phenomenon shows that under the higher preload, slip movement between the FRP and steel plates did not occur. Therefore, it can be concluded that for bolted reinforcement/repair structures, the use of a higher bolt preload can help to eliminate the performance difference of the overall component caused by interface treatment errors. That is to say, to ensure the performance safety of the bolted repair structure, it is advisable to increase the pre-tightening force of the bolts (preferably using high-strength pre-tightening bolts).

## 4. Conclusions

This study provides insights into the repairing behaviors of cracked steel plates based on bolted FRP plates through experiments and FE analysis. The results of this study reveal the repair effectiveness and the influence factors (i.e., pre-tightening forces and friction coefficients) of this repair method. The results highlight the importance of considering preloads and friction coefficients in the design of bolted repair for steel components. The following conclusions can be drawn:

1. By comparing the reference specimen (i.e., the pure cracked steel plate) and its corresponding repaired specimen (which was bolted with FRP plates), it was found that the bolted repairing method could significantly improve the tensile strengths of the cracked steel plates. Moreover, by examining the failure characteristics of the six specimens, composite failure modes—which included the cracking of the steel plate and bearing failure of the FRP—were observed in the repaired specimens.
2. The bolt repair of the FRP plates effectively mitigated the stress concentration at the crack tip in the specimen. As a consequence, the extreme stress level occurring near the tip following strengthening measures amounted to 238.68 MPa for the bolted specimen with a 2 mm width crack, which is significantly lower than that of the reference specimen (pure cracked specimen with a 2 mm width crack).
3. The discussion of the effect of the preloads of the bolts revealed that the pre-tightening force of bolts played a crucial role in influencing the stress magnitude within a specimen’s crack area. As the pre-tightening force increases, the stress levels within the crack area gradually trend upwards, and the change in the von Mises stress for node R can be modeled kinetically with a cubic function curve.
4. The mechanical performance of the specimen will be affected by the interface friction coefficient, and as the interface friction coefficient increases (from 0.3 to 0.7), the stress of node R gradually decreases accordingly. In addition, based on the discussion on the working conditions featuring higher preloads (such as when the preloads were 10,000 N), it was concluded that the use of a higher bolt preload can help to eliminate the performance difference of the overall component caused by interface treatment errors. It is worth noting that this study mainly studied the repairing/strengthening effects of bolted specimens of FRP and cracked steel plates; however, the relaxation effects of bolted structures under vibrational loads may result in different performances, and the influences of relaxation effects require additional investigation in future studies.

## Figures and Tables

**Table 1:** Mechanical properties of the GFRP, adhesive, and steel.

Mechanical Property	GFRP	Steel Plates	Steel Bolts
Young’s modulus (GPa)	20.22 (longitudinal direction) 12.39 (transversal direction) 13.66 (thickness direction)	203	202
Strength (MPa)	537.86 (longitudinal direction) 303.24 (transversal direction) 327.43 (thickness direction)	364 (yield strength) 542 (tensile strength)	645 (shear strength)
Poisson’s ratio	0.37	0.30	0.30

**Table 2:** Parameters for the specimens.

Specimen	a and b and c/mm	Tensile Strength/N	Failure Mode
Type I	40 and 20 and 1	1298	Cracking of steel plate +shear-out failure of FRP
Type II	40 and 20 and 2	1573	Cracking of steel plate +shear-out failure of FRP
Type III	40 and 20 and 3	1750	Cracking of steel plate +shear-out failure of FRP
Reference I	40 and 20 and 1	813	Cracking of steel plate
Reference II	40 and 20 and 2	1038	Cracking of steel plate
Reference III	40 and 20 and 3	1204	Cracking of steel plate

## Data Availability

Data will be made available on request.
